# MicroRNA-885-3p alleviates bronchial epithelial cell injury induced by lipopolysaccharide via toll-like receptor 4

**DOI:** 10.1080/21655979.2022.2032939

**Published:** 2022-02-14

**Authors:** Yahui Shen, Aigui Jiang, Rong Chen, Xiaoyan Gao, Guixian Song, Huiyu Lu

**Affiliations:** a Department of Respiratory and Critical Care Medicine, No. 5 Affiliated Hospital of Nantong University (Taizhou People’s Hospital), Taizhou, Jiangsu, China; b Department of Cardiology, No. 5 Affiliated Hospital of Nantong University (Taizhou People’s Hospital), Taizhou, Jiangsu, China

**Keywords:** Asthma, MiR-885-3p, TLR4, cell injury, NF-κB-MyD88 pathway

## Abstract

Airway inflammation is one of the typical pathological characteristics of asthma. MicroRNAs (miRNAs) play important roles in regulating inflammation. Nevertheless, miRNA-885-3p (miR-885-3p)’s role in asthmatic inflammation and the underlying mechanism need to be explained. In this work, miR-885-3p expression and toll-like receptor 4 (TLR4) expression in asthma patients’ plasma and lipopolysaccharide (LPS)-treated 16HBE cells were detected through quantitative real-time PCR. The interleukin-8 (IL-8), tumor necrosis factor-α (TNF-α) and interleukin-6 (IL-6) levels in 16HBE cell supernatant were examined via enzyme-linked immunosorbent assay. Cell counting kit-8 (CCK-8) assay and flow cytometry were employed to examine 16HBE cell viability and apoptosis, respectively. Western blotting was performed to examine the expression of TLR4, cleaved caspase-3, B-cell lymphoma-2 (Bcl-2), nuclear factor-kappa B (NF-κB) p65, Bcl-2-related X protein (Bax), phosphorylated (p)-NF-κB p65 and myeloid differentiation primitive-response protein 88 (MyD88) in 16HBE cells. Furthermore, the targeted relationship between TLR4 and miR-885-3p in 16HBE cells was determined through dual-luciferase reporter gene assay. Compared with healthy volunteers, miR-885-3p expression in acute asthma patients’ plasma was significantly downregulated. In 16HBE cells, the stimulation of LPS reduced miR-885-3p expression. MiR-885-3p overexpression reduced LPS-stimulated 16HBE cell injury by enhancing cell viability, and suppressing the levels of inflammatory factors and apoptosis. Furthermore, TLR4 was identified as miR-885-3p’s target gene. TLR4 overexpression weakened the impacts of miR-885-3p on LPS-stimulated cell injury and NF-κB-MyD88 signaling. In conclusion, miR-885-3p can reduce LPS-induced 16HBE cell damage, via targeting TLR4 to suppress the NF-κB-MyD88 pathway.

## Graphical abstract

**Figure:**
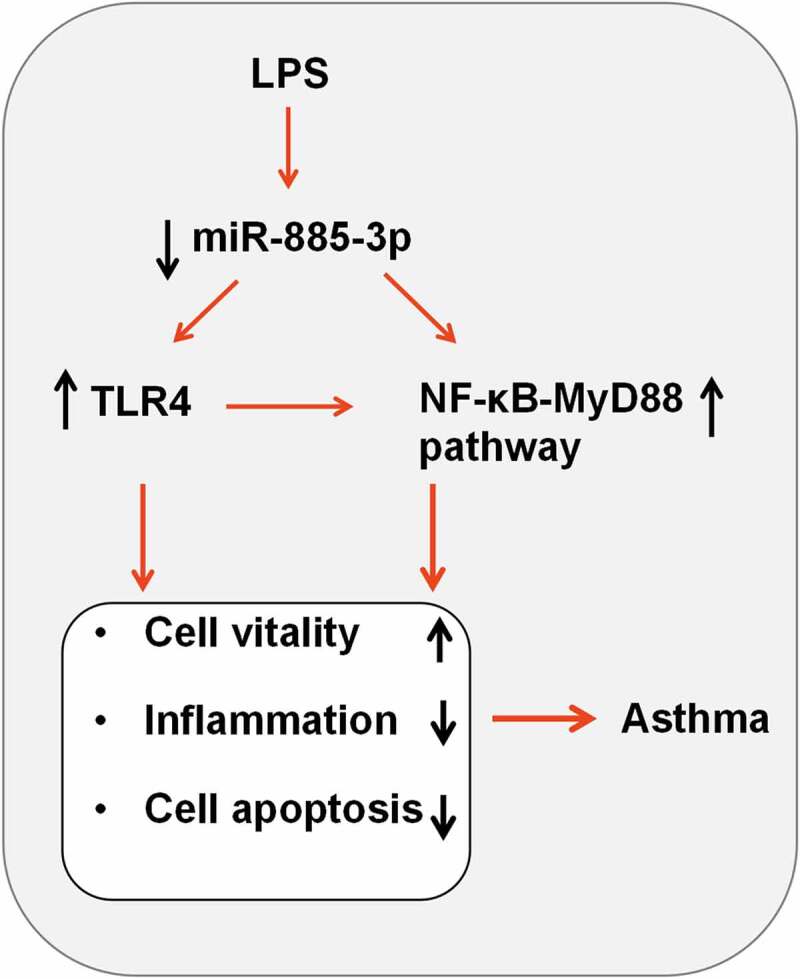

## 1. Introduction

The pathogenesis of bronchial asthma (abbreviated as asthma) involves multiple cells (including airway structural cells and inflammatory cells) and inflammatory factors [1]. About 300 million people worldwide suffer from asthma, posing a huge social and economic burden [2–4]. Respiratory epithelial cells are the key barrier of the respiratory tract [5]. Studying the mechanism underlying the inflammatory injury of airway epithelial cells is highly significant to improving the clinical asthma treatment.

As a type of small non-coding RNA of 18–23 nucleotides, microRNAs (miRNAs or miRs) bind to the target mRNA 3’ untranslated region (3ʹUTR) to modulate gene expression at post-transcriptional level [6,7]. Increasing evidence shows that aberrant miRNA expression is correlated with the pathogenesis of multiple human diseases such as pulmonary fibrosis, cancer and asthma [8–10]. For example, miR-885-3p enhances the docetaxel sensitivity of pulmonary adenocarcinoma cells by down-regulating Aurora A [11]; miR-885-3p expression is reduced in the type-1 diabetes patient’s peripheral blood monocytes, and miR-885-3p can restrain the pro-inflammatory cytokine production through targeting Toll-like receptor 4 (TLR4)/ nuclear factor-kappa B (NF-κB) signal transduction [12]. Nevertheless, so far, miR-885-3p’s role in asthmatic pathogenesis and its underlying mechanism is still unknown.

TLR4 is a kind of transmembrane protein in the plasma or endosomal membrane. It mediates immune inflammatory response, and participates in the pathogenesis of atherosclerosis, coronary heart disease, asthma and other diseases [13,14]. TLR4 activation induces NF-κB’s activation and nuclear translocation through multiple signal cascades, thereby promoting the secretion of interleukin-8 (IL-8), tumor necrosis factor-α (TNF-α) and interleukin-6 (IL-6) [15]. NF-κB is crucial to regulate immuno-inflammatory responses [16]. In addition, in the NF-κB activation process, myeloid differentiation primitive-response protein 88 (MyD88) is a crucial molecule [17]. Importantly, the TLR4/MyD88/NF-κB axis is reported to partake in asthma’s pathogenesis [18]. Nonetheless, the mechanism underlying TLR4/MyD88/NF-κB activation in asthma remains to be further explored.

As the main component of gram-negative bacterial endotoxin, lipopolysaccharide (LPS) can induce inflammatory response in nonimmune cells including airway epithelial cells, thereby causing cell inflammatory injury [19]. In the present work, it was hypothesized that miR-885-5p was a player in the inflammatory injury of airway epithelial cells during the pathogenesis of asthma. Using 16HBE treated with LPS as the cell model, this study was performed to explore the protective role of miR-885-3p against inflammatory damage of human bronchial epithelial cells and its potential molecular mechanism, and investigate the interaction between the TLR4/MyD88/NF-κB pathway and miR-885-3p.

## 2. Materials and methods

### 2.1. Bioinformatics analysis

The microarray dataset GSE25230 used in this study was obtained from the Gene Expression Omnibus (GEO) database (http://www.ncbi.nlm.nih.gov/gds/). The microarray data analysis was performed as previously described [19]. In short, the interactive web tool GEO2R (www.ncbi.nlm.nih.gov/geo/geo2r) was used to analyze the expression profile of miRNA in bronchial epithelial cells from healthy and asthmatic donors. MiRNAs with *P* < 0.05 and log2 (fold change)> 1 or <-1 were considered to be significantly differentially expressed.

### 2.2. Clinical samples

All patients, healthy volunteers, and guardians of the participants offered an informed consent, and our study got the approval from the Ethics Committee of No. 5 Affiliated Hospital of Nantong University. All patients had no cancer, hypertension, diabetes, infectious diseases and other cardiovascular and cerebrovascular diseases. From March 2018 to August 2019, blood samples were collected from 35 acute asthma patients who were treated in No. 5 Affiliated Hospital of Nantong University, and the blood samples from 35 healthy individuals were collected as controls. The patients received anti-inflammatory treatment, antibiotic therapy and glucocorticoid therapy, accompanied with oxygen therapy. The controls are healthy subjects who received physical examination in the hospital. The pulmonary function of the healthy controls was normal. There were no significant differences of the gender, age, body mass index and other characteristics between the asthma patients and the healthy controls. The collected peripheral blood sample (5 ml) was kept in ethylene diamine tetraacetic acid (EDTA)-containing test tubes. The blood samples were centrifuged at room temperature at 1500 rpm for 10 min to isolate the plasma, and plasma samples were maintained at −80°C for subsequent research.

### 2.3. Cell culture and LPS treatment

From the Chinese Academy of Sciences Cell Bank (Shanghai, China), human bronchial epithelial cells (16HBE) were bought. 10% (*v*/*v*) fetal bovine serum (Gibco; Thermo Fisher Scientific, Inc., Waltham, MA, USA) and 0.1 mg/ml streptomycin and 100 U/ml penicillin (Invitrogen, Carlsbad, CA, USA) were added to the Dulbecco’s modified Eagle’s medium (Hyclone, Logan, UT, USA). The medium mentioned above was utilized to culture 16HBE cells in 5% CO_2_ at 37°C in a humidified environment. To establish the [20]* injury model, the cells were treated by different concentrations (5, 10 and 20 μg/ml) of LPS (Sigma-Aldrich, St Louis, MO, USA) for 12 h which were added to the medium [21].

### 2.4. Cell transfection

From GenePharma (Shanghai, China), mimics negative control (miR-NC) and miR-885-3p mimics were purchased. To construct the TLR4 overexpression vector, the TLR4 sequence was integrated into pcDNA 3.1 vector (Invitrogen, Carlsbad, CA, USA). Lipofectamine® 3000 (Invitrogen, Carlsbad, CA, USA) was employed, under the manufacturer’s protocol, to conduct cell transfection. After the transfection, the cells were cultured for 24 h at 37°C in 5% CO_2_ for further experiments.

### 2.5. Quantitative real-time PCR (qRT-PCR)

TRIzol reagent (Invitrogen, Carlsbad, CA, USA) was employed for the extraction of the total RNA from the plasma and cells, which was subsequently synthesized by the PrimeScript™ RT kit (Takara, Dalian, China) into first-strand cDNA. The FastStart Universal SYBR Green Master Mix (Roche, Basel, Switzerland) was adopted to conduct qRT-PCR on the ABI 7500 system (Applied Biosystems, Foster City, CA, USA) following the manufacturer’s instructions. TLR4 mRNA expression and miR-885-3p expression were normalized to glyceraldehyde-3-phosphate dehydrogenase (GAPDH) and U6 small nuclear RNA (U6), respectively, and the relative expression was calculated applying the 2^−ΔΔCT^ method [22]. The primer sequences (F, Forward; R, Reverse): TLR4, F: 5’-AACTCTGGATGGGGTTTCCT-3’, R: 5’-ACAACCTCCCTTCTCAACC-3’; miR-885-3p, F: 5’-GAGCACGAGGCAGTAGGCAAAGTGT-3’, R: 5’-GAGGCAGCGGGGTGTAGTGGATAGA-3’; U6, F: 5’-CTCGCTTCGGCAGCACATATACTA-3’, R: 5’-ACGAATTTGCGTGTCATCCTTGC-3’; GAPDH, F: 5’-CATCCCTTCTCCCCACACACAC-3’, R: 5’-AGTCCCAGGGCTTTGATTTG-3’.

### 2.6. Enzyme-linked immunosorbent assay (ELISA)

16HBE cells were inoculated at 1 × 10^5^ cells/well into 6-well plates. After 48 h of culturing, the cells and medium were harvested and centrifuged at 4°C at 1000 × g for 10 min. After that, the supernatant after centrifugation was harvested. Ultimately, the corresponding ELISA kits (TaKaRa, Dalian, China) were employed for detecting the content of IL-8, TNF-α and IL-6 under the manufacturer’s protocol.

### 2.7. Cell viability experiment

The transfected 16HBE cells were transferred to 96-well plates (5000 cells/well), and LPS (10 μg/ml) was employed for stimulating the cells for 12 h. Subsequently, according to the manufacturer’s instructions, the cell viability was evaluated using the Cell Counting Kit-8 (CCK-8; MedChemExpress, Monmouth Junction, NJ, USA). After treatment with LPS, the cells in each well was added with CCK-8 solution (10 μL), and the cells were cultured for 1 h at 37°C. Next, the absorbance of the cells was measured at 450 nm using a microplate reader (Bio-Rad, Hercules, CA, USA).

### 2.8. Flow cytometry

Following transfection, the cells were treated with LPS for 12 h. Subsequently, the cells were collected, rinsed with phosphate buffered solution (PBS) twice, and then resuspended in binding buffer (100 μL). Subsequently, 5 μL of Annexin V-fluorescein isothiocyanate (FITC)/propidium iodide (PI) staining solution was added to stain the cells. After culturing the cells for 15 min away from light, the cell apoptosis was examined with a FACScan flow cytometer (BD Bioscience, Frankin Lakes, NJ, USA).

### 2.9. Dual-luciferase reporter gene assay

The online StarBase software (http://starbase.sysu.edu.cn/) was used to predict the binding sites of miR-885-3p and TLR4 3’-UTR [23]. To synthesize reporter vectors pGL3-TLR4-MUT-3’-UTR or pGL3-TLR4-WT-3’-UTR, the 3’-UTR of TLR4 with miR-885-3p mutant (MUT) or wild-type (WT) binding site was amplified, and integrated into the pGL3 vector (Promega, Madison, WI, USA). Lipofectamine® 3000 reagent (Invitrogen, Carlsbad, CA, USA) was adopted to co-transfect miR-885-3p mimics or negative control (miR-NC) and luciferase reporter vectors into 16HBE cells. The Dual-Luciferase Reporter Assay System (Promega, Madison, WI, USA) was utilized 48 h after the transfection to detect the luciferase activity the cells following the manufacturer’s instruction.

### 2.10. Western blot assay

The total protein was isolated employing radio-immunoprecipitation assay (RIPA) lysis buffer (Beyotime, Shanghai, China) as previously reported [24]. Then, the protein separation was performed via sodium dodecyl sulfate-polyacrylamide gel electrophoresis, and the protein was transferred to a polyvinylidene difluoride (PVDF) (Millipore, Bedford, MA, USA) membrane. After blocking the membrane with 5% skim milk for 2 h at room temperature, the membrane and primary antibodies were incubated at 4°C overnight. The primary antibodies include anti-TLR4 (1:1000, ab13556, Abcam, Shanghai, China), anti-NF-κB p65 (1:1000, ab239882, Abcam), anti-MyD88 (1:1000, ab219413, Abcam), anti-phospho-NF-κB (p-NF-κB) p65 (Ser536) (1:1000, ab239882, Abcam) and anti-GAPDH (1:1000, ab9485, Abcam). Then the membranes and HRP-conjugated secondary antibody (1:1000, ab97051, Abcam) were incubated for 1 h at room temperature. Ultimately, the protein bands were developed by a chemiluminescence detection system (Millipore, Billerica, MA, USA), and the Quantity One software (Bio-Rad, Hercules, CA, USA) was utilized to quantify the protein bands.

### 2.11. Statistical analysis

Each experiment was conducted at least in triplicate. SPSS 20.0 software (IBM, Chicago, IL, USA) was the tool for statistical analysis. Mean ± standard deviation was the expression form of the data. Student’s *t*-test was used to make the comparison. When *P* < 0.05, a difference was of statistical significance.

## 3. Results

The current study explored the expression of miR-885-3p in acute asthma patients’ plasma and LPS-stimulated 16HBE cells. Then, the role and mechanism of miR-885-3p in regulating the viability, inflammation and apoptosis of of LPS-stimulated 16HBE cells was investigated through *in vitro* experiments. Furthermore, the interaction between miR-885-3p and TLR4/MyD88/NF-κB pathway was studied. In summary, our study showed that miR-885-3p inhibited LPS-induced bronchial epithelial cell inflammatory damage by regulating the TLR4/MyD88/NF-κB pathway.

### 3.1. MiR-885-3p is downregulated in asthma patients’ plasma and LPS-induced 16HBE cells

**Figure 1.**
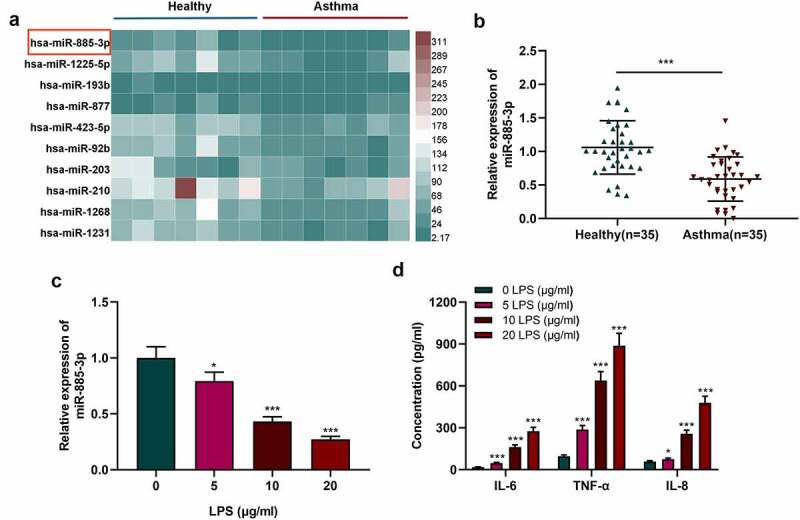
MiR-885-3p is low-expressed in asthma patients’ plasma and 16HBE cells stimulated by LPS.

By analyzing the microarray dataset (GSE25230), it was revealed that miR-885-3p was underexpressed in bronchial epithelial cells in asthma as against the control (Figure 1(a)). qRT-PCR was carried out to examine miR-885-3p expression in the plasma of 35 acute asthma patients and 35 healthy subjects. It was suggested that miR-885-3p level in the serum of asthmatic patients was markedly down-regulated compared with healthy volunteers (Figure 1(b)). Additionally, to construct an *in-vitro* cell injury model, different concentrations (5, 10, and 20 μg/ml) of LPS were utilized to treat 16HBE cells. qRT-PCR indicated that miR-885-3p expression was suppressed in 16HBE cells with the treatment of LPS in a dose-dependent manner (Figure 1(c)). ELISA indicated that LPS treatment remarkably enhanced the secretion of pro-inflammatory cytokines (IL-8, IL-6 and TNF-α) in 16HBE cells (Figure 1(d)). Since 10 μg/ml LPS could significantly reduce miR-885-3p expression in 16HBE cells and increase inflammatory cytokine secretion, 10 μg/ml LPS was selected for follow-up experiments.

(a) The heat map shows the differential expression of miRNAs in normal and asthmatic bronchial epithelial cells.(b,c) Detection of miR-885-3p expression in asthma patients’ plasma and 16HBE cells stimulated by LPS via qRT-PCR.(d) ELISA was performed to detect the content of TNF-α, IL-8, and IL-6 in LPS-stimulated 16HBE cell supernatant.All of the experiments were performed in triplicate. Student’s t test, *P < 0.05, and ***P < 0.001.

### 3.2. Overexpression of miR-885-3p alleviates LPS-induced cellular inflammatory response

**Figure 2.**
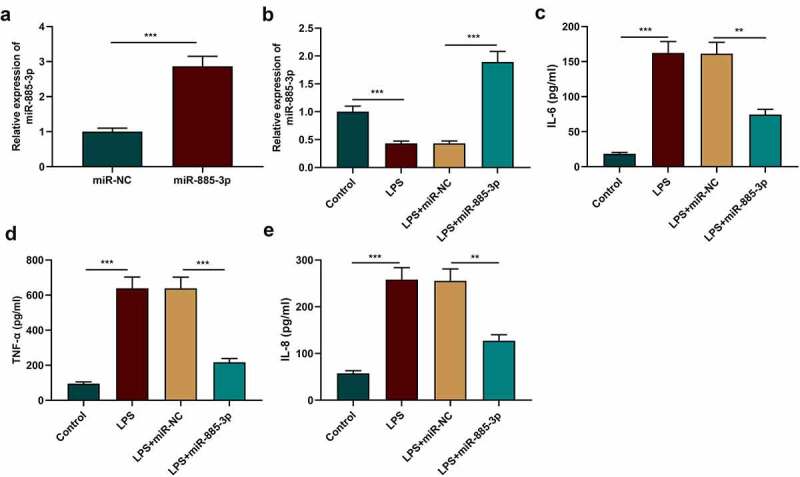
MiR-885-3p alleviates LPS-stimulated 16HBE cell inflammation.

To study miR-885-3p’s functions in LPS-induced inflammatory response, miR-NC or miR-885-3p mimics were transfected into 16HBE cells, followed by the verification of the transfection efficiency via qRT-PCR. It was validated that the transfection of miR-885-3p mimics significantly reduced miR-885-3p expression in 16HBE cells (Figure 2(a)). Additionally, the transfection of miR-885-3p mimics counteracted the suppressive effect of LPS stimulation on miR-885-3p expression (Figure 2(b)). ELISA was performed for detecting the IL-8, IL-6 and TNF-α levels in the cell culture supernatant, and it was revealed that miR-885-3p overexpression reduced the IL-8, TNF-α and IL-6 content in 16HBE cells (Figure 2(c–e)). The aforementioned findings suggest that miR-885-3p counteracts LPS-induced bronchial epithelial cell inflammation.

(a) Detection of miR-885-3p expression in 16HBE cells transfected with miR-NC or miR-885-3p mimics via qRT-PCR.(b) qRT-PCR was performed to detect the relative miR-885-3p expression in 16HBE cells after transfection of miR-885-3p mimic or stimulated with 10 μg/ml LPS.(c–e) ELISA was performed to measure IL-6, TNF-α and IL-8 concentrations in the supernatant of 16HBE cells stimulated with 10 μg/ml LPS or transfected with miR-885-3p mimic.All of the experiments were performed in triplicate. Student’s t test, **P < 0.01 and ***P < 0.001.

### 3.3. MiR-885-3p reduce LPS-induced 16HBE cell damage

**Figure 3.**
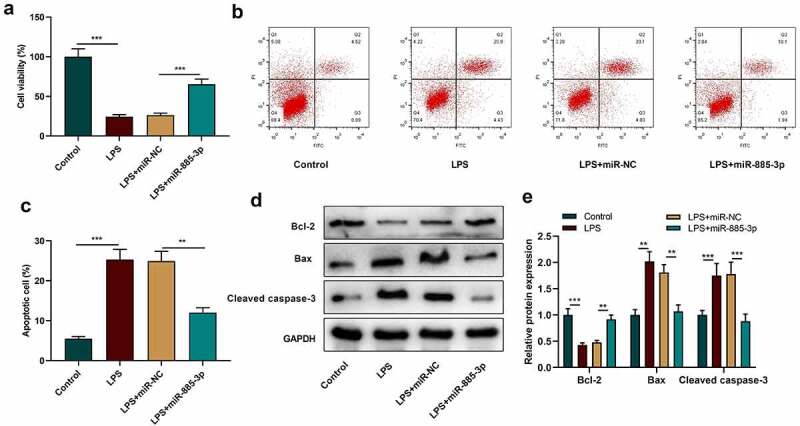
The impacts of miR-885-3p on 16HBE cell viability and apoptosis.

To further explain miR-885-3p’s functions in LPS-induced inflammatory damage, CCK-8 was adopted to evaluate 16HBE cell viability after transfecting miR-885-3p mimic. It was manifested that in contrast with the control group, LPS treatment markedly suppressed the viability of 16HBE cells, while the transfection of miR-885-3p mimics reversed this inhibiting effect (Figure 3(a)). Next, flow cytometry was conducted to examine 16HBE cell apoptosis, and it was revealed that LPS stimulation markedly facilitated 16HBE cell apoptosis, but miR-885-3p overexpression dramatically abated the promoting effect of LPS stimulation on the apoptosis (Figure 3(b,c)). Moreover, Western blot indicated that, compared with the control, the Bcl-2 protein level in the LPS treatment group was markedly reduced, yet cleaved caspase-3 expression and Bax expression were enhanced (Figure 3(d,e)); as against the LPS+miR-NC group, the transfection of miR-885-3p mimics promoted Bcl-2 expression, yet repressed the cleaved caspase-3 and Bax levels (Figure 3(d,e)). The above-mentioned findings further support that miR-885-3p can relieve the inflammatory injury of 16HBE cells induced by LPS.

(a–c) CCK-8 assay and flow cytometry were conducted to evaluate the viability (A) and apoptosis rate (B, C) of 16HBE cells transfected with miR-885-3p mimic or treated with 10 μg/ml LPS, respectively.(d,e) Detection of the cleaved caspase-3, Bax and Bcl-2 levels in 16HBE cells, which were stimulated with 10 μg/ml LPS or transfected with miR-885-3p mimic, via Western blotting.All of the experiments were performed in triplicate. Student’s t test, **P < 0.01, and ***P < 0.001.

### 3.4. MiR-885-3p directly targets TLR4

**Figure 4.**
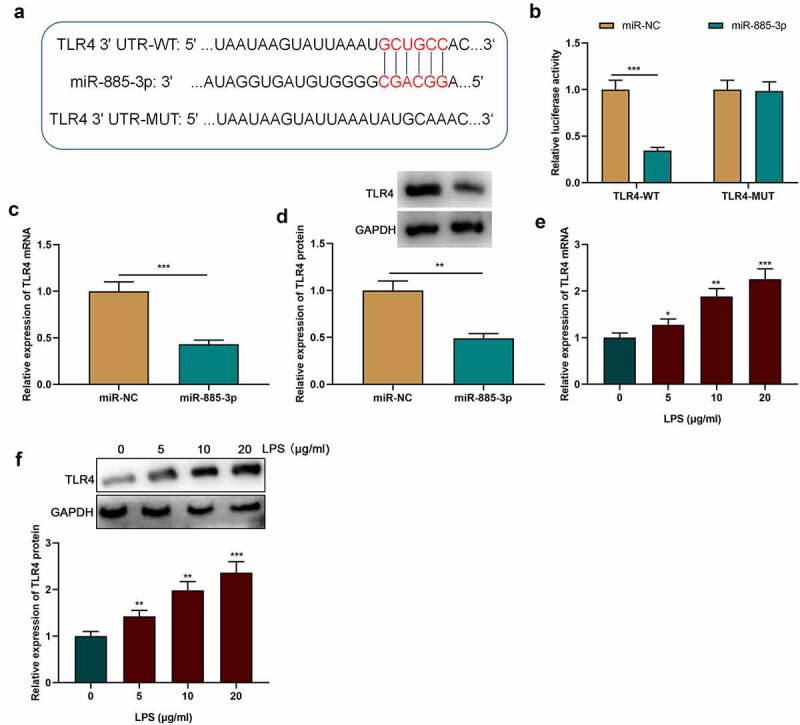
TLR4 is miR-885-3p’s downstream target.

Previous research has shown that TLR4-mediated inflammation is associated with asthma’s pathogenesis [19]. Therefore, TLR4 was selected for follow-up analysis. StarBase online database (http://starbase.sysu.edu.cn/) was searched to predict the binding site between miR-885-3p and TLR4 mRNA 3’-UTR (Figure 4(a)). Dual-luciferase reporter assay showed that in comparison with the control group, the transfection of miR-885-3p mimics suppressed the luciferase activity of the TLR4-WT vector in 16HBE cells, yet failed to observably influence that of the TLR4-MUT vector (Figure 4(b)). Furthermore, compared with the miR-NC group, the transfection of miR-885-3p mimics dramatically reduced TLR4 mRNA and protein expression in 16HBE cells (Figure 4(c,d)). Additionally, it was unveiled that TLR4 mRNA and protein were significantly up-regulated in LPS-stimulated 16HBE cells in a concentration-dependent manner (Figure 4(e,f)). The aforementioned evidence suggests that TLR4 is a downstream miR-885-3p target.

(a) The binding sequence of TLR4 mRNA 3’-UTR with miR-885-3p.(b) The targeted relationship of miR-885-3p with TLR4 was verified through dual-luciferase assay.(c,d) Western blot and qRT-PCR were utilized for detecting TLR4 mRNA and protein expression in 16HBE cells with transfection of miR-885-3p mimic.(e,f) Western blotting and qRT-PCR were conducted to detect TLR4 mRNA and protein expression in 16HBE cells stimulated with LPS at different concentrations (5, 10, and 20 μg/ml).All of the experiments were performed in triplicate. Student’s t test, *P < 0.05, **P < 0.01, and ***P < 0.001.

### 3.5. MiR-885-3p regulates LPS-caused cell inflammatory injury via targeting TLR4

**Figure 5.**
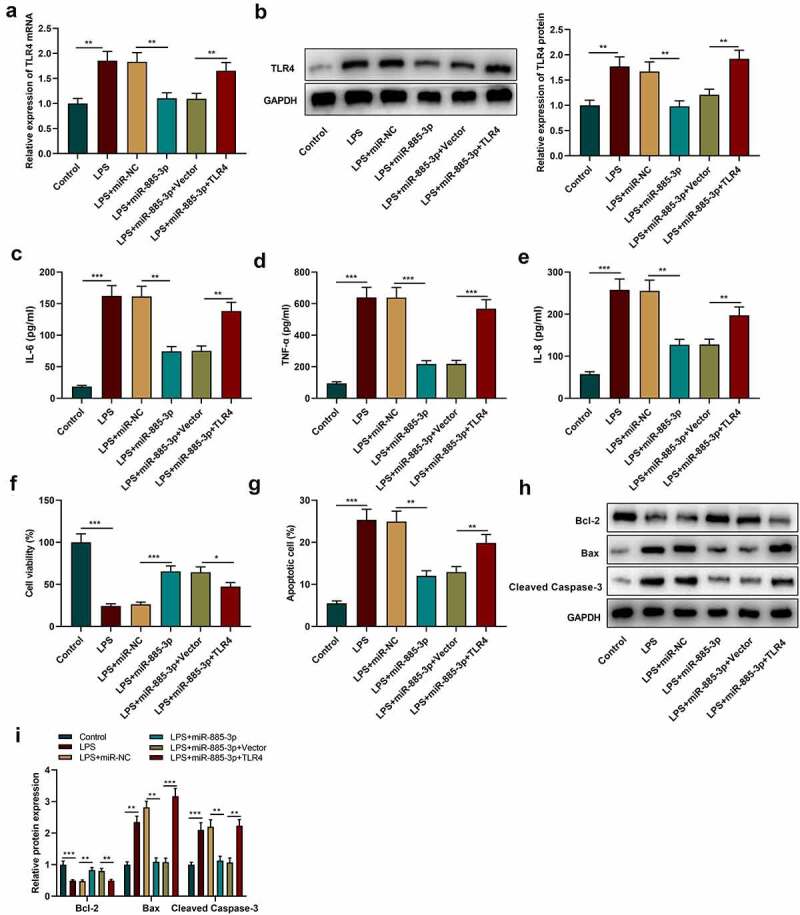
MiR-885-3p suppresses LPS-induced cell inflammatory injury through targeting TLR4.

To further examine the role of miR-885-3p/TLR4 axis in LPS-caused cell inflammatory damage, TLR4 overexpression plasmids or empty plasmids were transfected into 16HBE cells with miR-885-3p mimics. Subsequently, TLR4 expression was examined through Western blotting and qRT-PCR, and it was revealed that the transfection of miR-885-3p mimics counteracted the promotional effect of LPS stimulation on TLR4 mRNA and protein expression compared with the LPS+miR-NC group (Figure 5(a,b)); compared with the LPS+miR-885-3p+Vector group, TLR4 mRNA and protein expression levels were observably increased in the cells of the LPS+miR-885-3p+TLR4 group (Figure 5(a,b)). ELISA was then conducted for detecting the IL-8, TNF-α and IL-6 levels in 16HBE cell culture supernatant, and it was demonstrated that TLR4 overexpression abated the inhibiting effect of miR-885-3p mimics on the production of IL-6, TNF-α and IL-8 (Figure 5(c,e)). CCK-8 experiment indicated that the promoting effect of miR-885-3p overexpression on cell viability could be weakened by TLR4 overexpression (Figure 5(f)). Furthermore, it was found that TLR4 overexpression attenuated the inhibiting effect of miR-885-3p mimics on LPS-induced 16HBE cell apoptosis (Figure 5(g)). Moreover, in comparison to the LPS+miR-885-3p+Vector group, Bcl-2 protein expression in the cells of the LPS+miR-885-3p+TLR4 group was decreased, while cleaved caspase-3 expression and Bax expression were enhanced (Figure 5(h,i)). The aforementioned findings imply that miR-885-3p can reduce 16HBE cell inflammation and injury induced by LPS by targeting TLR4.

(a,b) Western blotting and qRT-PCR were conducted to examine TLR4 mRNA and protein expression in 16HBE cells with transfection of miR-885-3p mimic or stimulated with 10 μg/ml LPS or co-transfected with TLR4 overexpression plasmid and miR-885-3p mimic.(c–e) ELISA was performed to detect TNF-α, IL-8 and IL-6 levels in 16HBE cell culture supernatant.(f,g) 16HBE cell viability (F) and apoptosis rate (G) were evaluated via CCK-8 and flow cytometry.(h,i) Cleaved caspase-3, Bax and Bcl-2 expression levels in 16HBE cells were detected by Western blotting.All of the experiments were performed in triplicate. Student’s t test, *P < 0.05, **P < 0.01, and ***P < 0.001.

### 3.6. MiR-885-3p modulates NF-κB-MyD88 pathway in 16HBE cells

**Figure 6.**
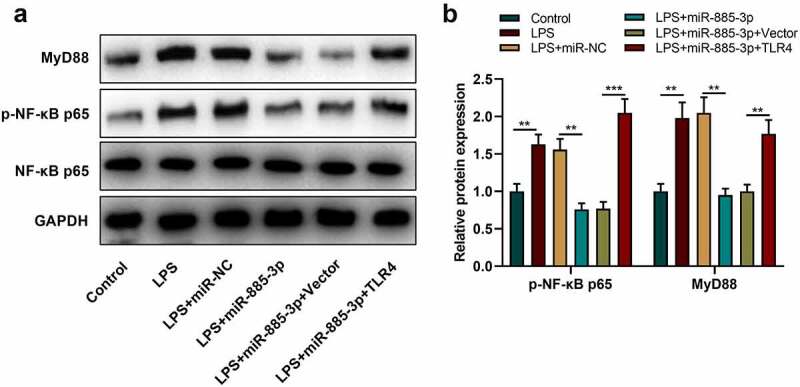
MiR-885-3p suppresses the NF-κB-MyD88 signaling activation.

Reportedly, the TLR4-NF-κB-MyD88 axis is implicated in LPS-stimulated inflammation [17,18]. Therefore, to decipher the relationship between NF-κB-MyD88 pathway and miR-885-3p in LPS-induced inflammation, phosphorylated (p)-NF-κB p65 expression and MyD88 expression were examined by Western blot. It was demonstrated that p-NF-κB p65 and MyD88 protein expression in 16HBE cells after LPS stimulation were dramatically enhanced compared with the control group; in comparison with the LPS+miR-NC group, after LPS stimulation, MyD88 and p-NF-κB p65 were remarkably downregulated in the cells transfected with miR-885-3p mimic, whereas TLR4 overexpression could reverse the inhibiting effect that the miR-885-3p mimic transfection had on MyD88 expression and p-NF-κB p65 expression (Figure 6(a,b)). The above-mentioned findings suggest that miR-885-3p can modulate the NF-κB-MyD88 axis via targeting TLR4 to inhibit the inflammation of bronchial epithelial cells.

(a,b) Western blotting was utilized for detecting p-NF-κB p65, MyD88 and NF-κB p65 expression in 16HBE cells with transfection of miR-885-3p mimics or treated with 10 μg/ml LPS or co-transfected with TLR4 overexpression plasmid and miR-885-3p mimic.All of the experiments were performed in triplicate. Student’s t test, *P < 0.05, and **P < 0.01.

## 4. Discussion

Asthma’s pathogenesis is correlated with airway hyperresponsiveness, airway inflammation, and airway remodeling, accompanied by airway epithelial cell changes and mucous gland hyperplasia, airway smooth muscle cell migration and proliferation, etc [25,26]. Mounting evidence shows that inflammation is pivotal in asthma’s pathogenesis, and suppressing inflammation can reduce the severity of asthma [27]. LPS can regulate Th2-type immune response, and stimulate the inflammation of multiple cells through facilitating pro-inflammatory cytokine expression [28]. It has been reported that the synergy of LPS and poly-L-arginine can facilitate the secretion of IL-8 and IL-6 in NCI-H292 cells by activating the JNK signal pathway [29]. In addition, metformin promotes the inflammatory damage of airway epithelial cells caused by LPS stimulation by inhibiting NF-κB signaling, thereby reducing airway inflammation [5]. In this study, it was found that LPS stimulation promoted the secretion of inflammatory factors (IL-8, IL-6 and TNF-α) in 16HBE cell, and LPS stimulation could also induce cell apoptosis, which are consistent with the previous reports [5,28,29].

MiRNAs are pivotal in modulating cellular processes, for instance, cell growth, apoptosis, differentiation, and autophagy [30,31]. For instance, in the LPS-induced acute lung injury mouse model, miR-34b-5p inhibition alleviates pulmonary inflammation and apoptosis via targeting PGRN [32]; the transfection of miR-340 mimics suppresses pro-inflammatory cytokine secretion to improve the anti-inflammatory impact of dexmedetomidine in LPS-treated microglia BV2 [33]. Importantly, miRNAs have also been found to take part in asthma pathogenesis. For example, in asthmatic children, miR-29c is down-regulated in blood monocytes, and the miR-29c/B7-H3 axis can regulate acute allergic asthma attack by modulating Th2/Th17 cell differentiation [34]; another study reports that miR-192-5p relieves asthmatic airway remodeling and autophagy through targeting ATG7 and MMP-16 [35]. MiR-885-3p has an important effect on inflammatory response regulation: reportedly, miR-885-3p inhibits the secretion of IL-1β, IL-6 and TNF-α in THP-1 cells [13]. The current study revealed that miR-885-3p was down-regulated in acute asthma patients’ plasma and LPS-stimulated 16HBE cells. MiR-885-3p mimics can relieve the injury of LPS-induced 16HBE cells and repress inflammatory response, suggesting it is a protective factor during the pathogenesis of asthma.

Toll-like receptors (TLRs), known as a kind of pattern recognition receptor (PRR), are a key player of the innate immune system [36]. TLR4 is the first discovered member of the TLR family, and it is also a modulator in multiple pathological processes such as lipid metabolism disorders, immune inflammatory reactions, and oxidative stress [37,38]. MyD88 is an important adaptor molecule for TLR4 to regulate inflammatory response. TLR4 can activate the downstream transcription factor NF-κB through MyD88, and NF-κB translocation further triggers inflammatory response [39,40]. Some miRNAs have been found to participate in regulating TLR4/NF-κB/MyD88 signaling [41,42]. For instance, miR-27a inhibits inflammation in an acute lung injury mice model through blocking TLR4/NF-κB/MyD88 signaling [41]; another study reports that miR-140-5p retrains the NF-κB/MyD88 pathway via targeting TLR4, thereby suppressing 16HBE cell inflammation induced by PM2.5 [42]. A lot of studies support that the activation of TLR4/NF-κB/MyD88 pathway is associated with the excessive production of pro-inflammatory factors including IL-4, IL-5, IL-6, IL-13, TNF-α and so on, in human cells [43,44]. In our study, it was demonstrated that TLR4 was a target of miR-885-3p in 16HBE cells. It was also found that TLR4 overexpression can counteract the biological effects of miR-885-3p on 16HBE cells. Moreover, it was discovered that the transfection with miR-885-3p mimics suppressed p-NF-κB p65 expression and MyD88 expression in LPS-stimulated 16HBE cells, and this effect could be counteracted by TLR4 overexpression. The above-mentioned findings indicate that miR-885-3p is implicated in bronchial epithelial cell inflammation and damage by modulating the TLR4/NF-κB/MyD88 axis.

## 5. Conclusion

To sum up, this study reports that miR-885-3p suppresses NF-κB-MyD88 signaling by targeting TLR4 to relieve bronchial epithelial cells’ inflammatory response and injury. The current study offers new insights into understanding miR-885-3p’s role in asthma pathogenesis. This study indicates that miR-885-3p may be a biomarker to evaluate the severity of asthmatic patients, which remains to be validated with a larger number of patients. Additionally, our study suggests that, the drugs, which can up-regulate the expression of miR-885-3p in respiratory epithelium, can be potential alternative drugs to treat asthmatic patients, especially for who are insensitive to glucocorticoid therapy.

## Supplementary Material

Supplemental Material

## Data Availability

The data for supporting the findings of the present study are available upon request from the corresponding authors.

## Highlights

1. MiR-885-3p is lowly expressed in asthma patients’ plasma and LPS-stimulated 16HBE cells.
2. MiR-885-3p reduces inflammatory injury of respiratory epithelial cells caused by LPS stimulation.
3. MiR-885-3p can directly target and negatively regulate TLR4.
