# Supplementary material for: MicroRNA-885-3p alleviates bronchial epithelial cell injury induced by lipopolysaccharide via toll-like receptor 4
Source: Bioengineered. 2022 Feb 14;13(3):5305–17. doi: 10.1080/21655979.2022.2032939 (PMC8974227; doi:10.1080/21655979.2022.2032939)

**All original blots of three repeats**

Figure 3D The protein levels of Bcl-2, Bax and cleaved caspase-3 in 16HBE cells treated with 10 μg/ml LPS or transfected with miR-885-3p mimics.

Bcl-2 (01) Bcl-2 (02) Bcl-2 (03)


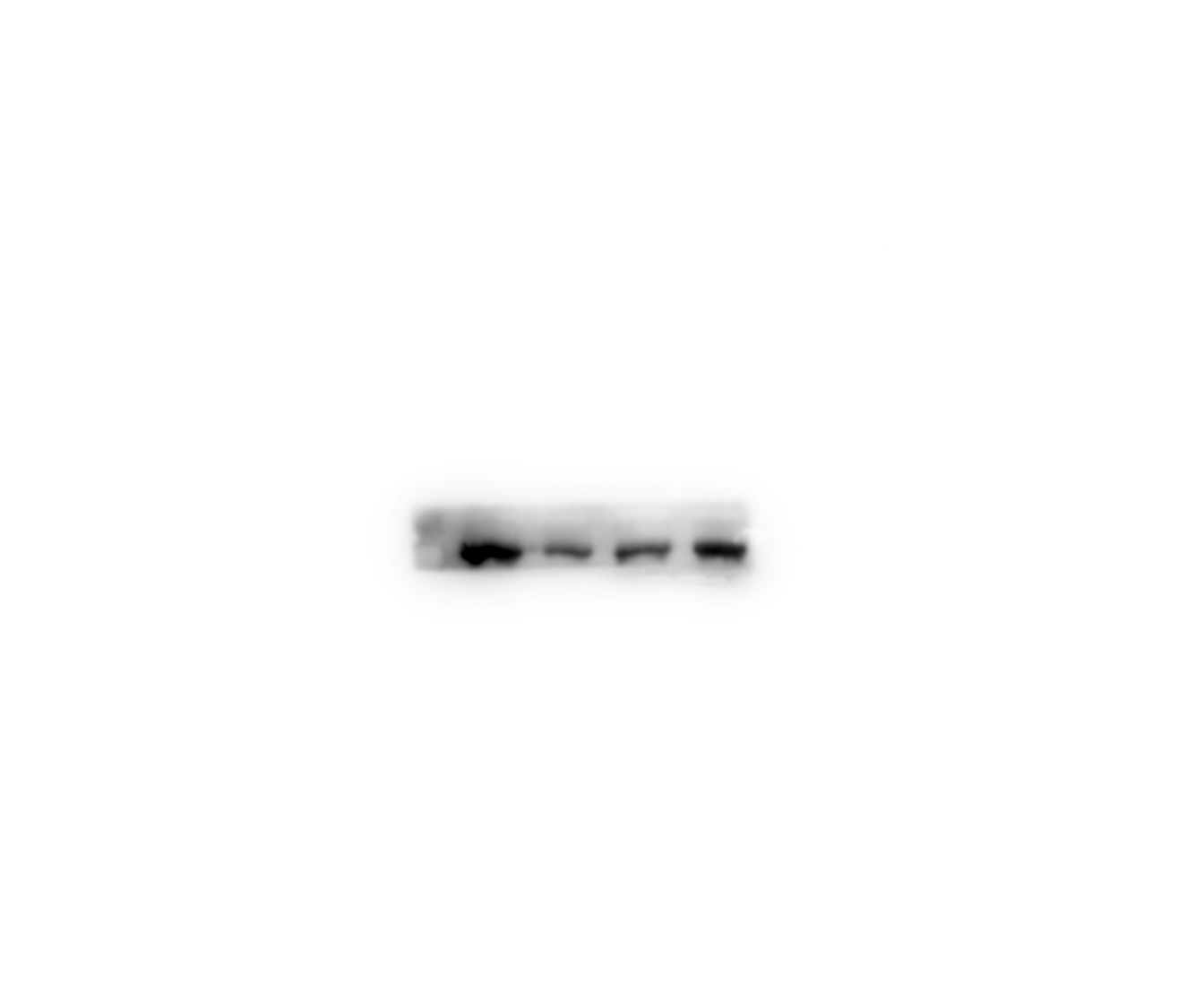

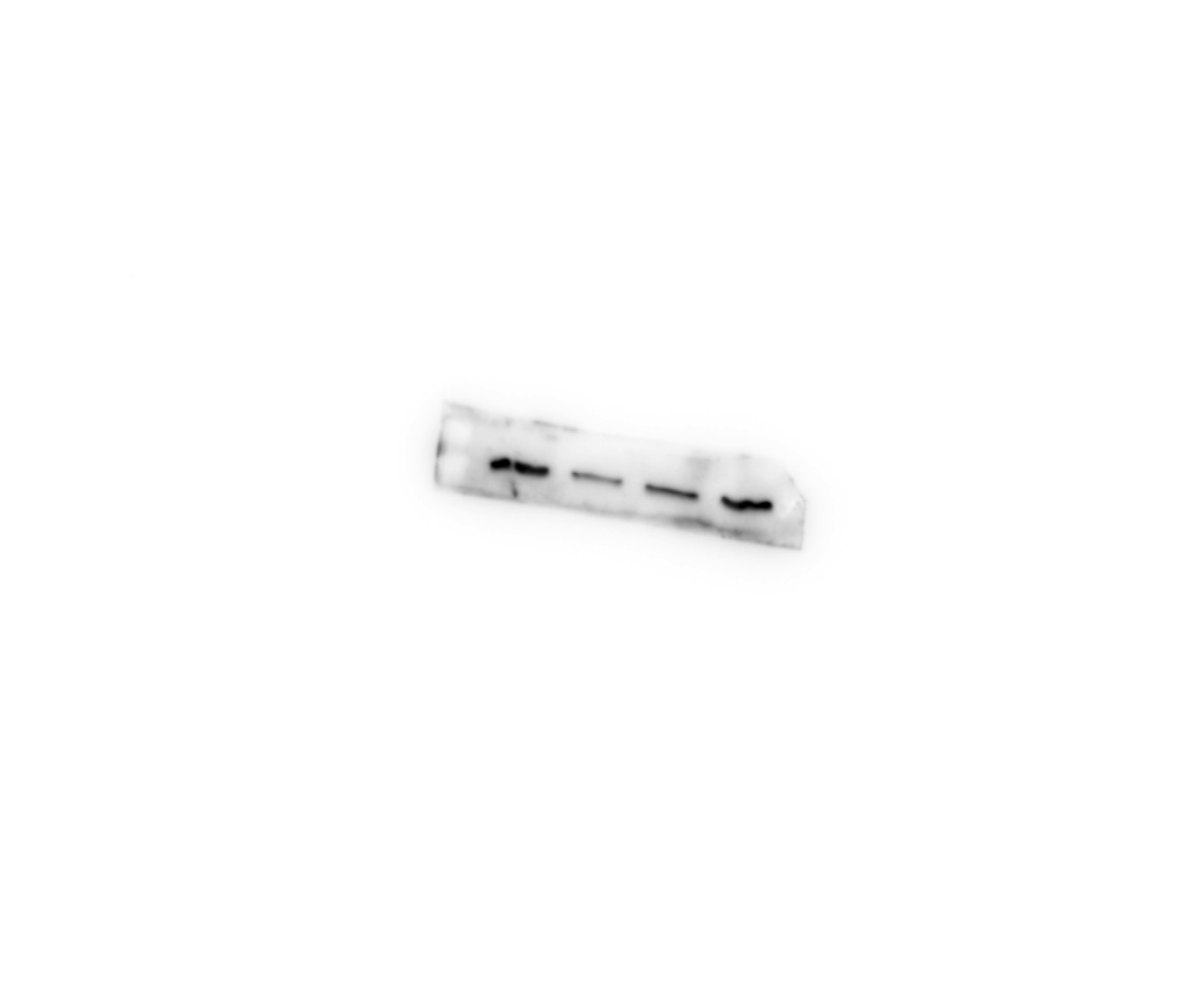

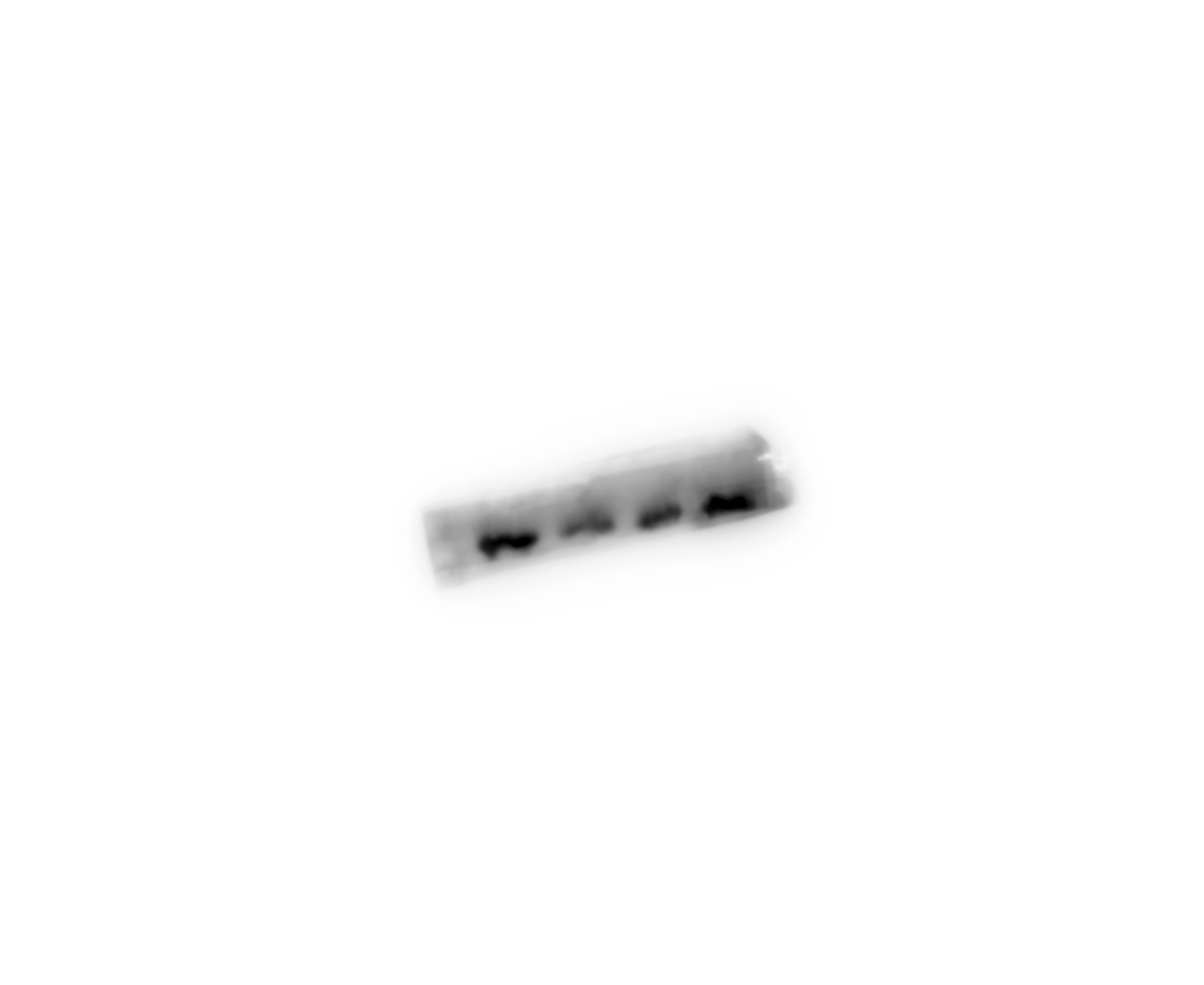


Bax (01) Bax (02) Bax (03)


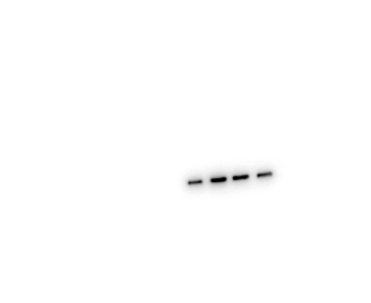

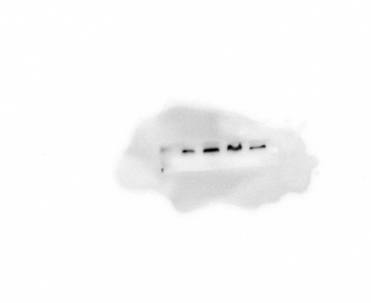

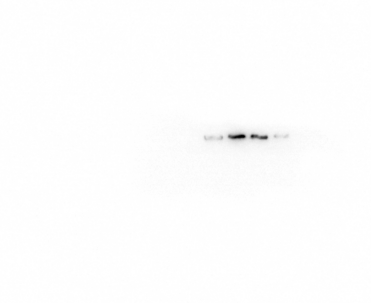


cleaved caspase-3 (01) cleaved caspase-3 (02) cleaved caspase-3 (03)


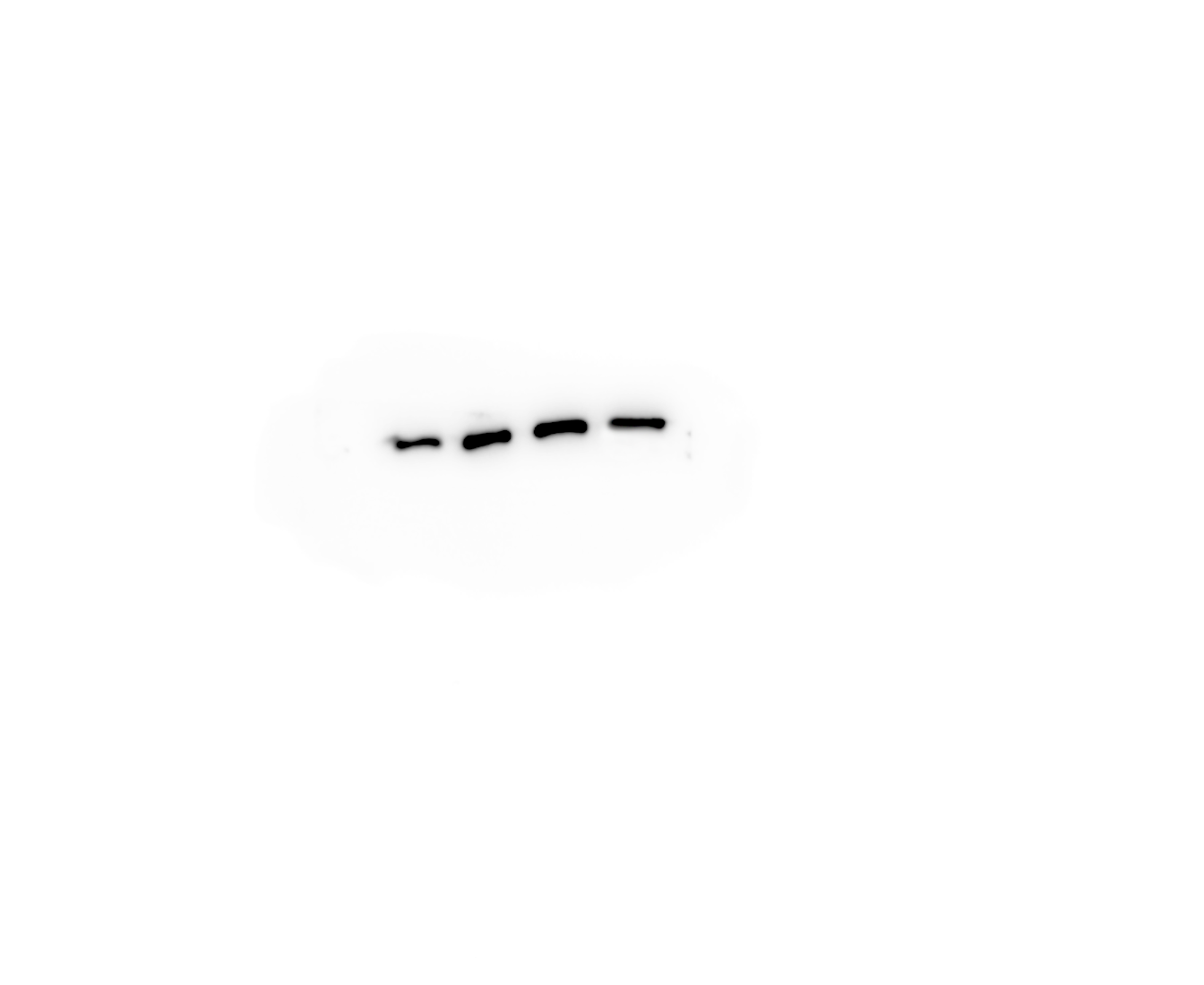

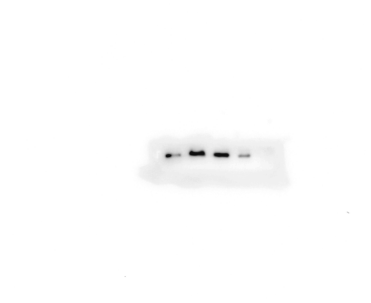

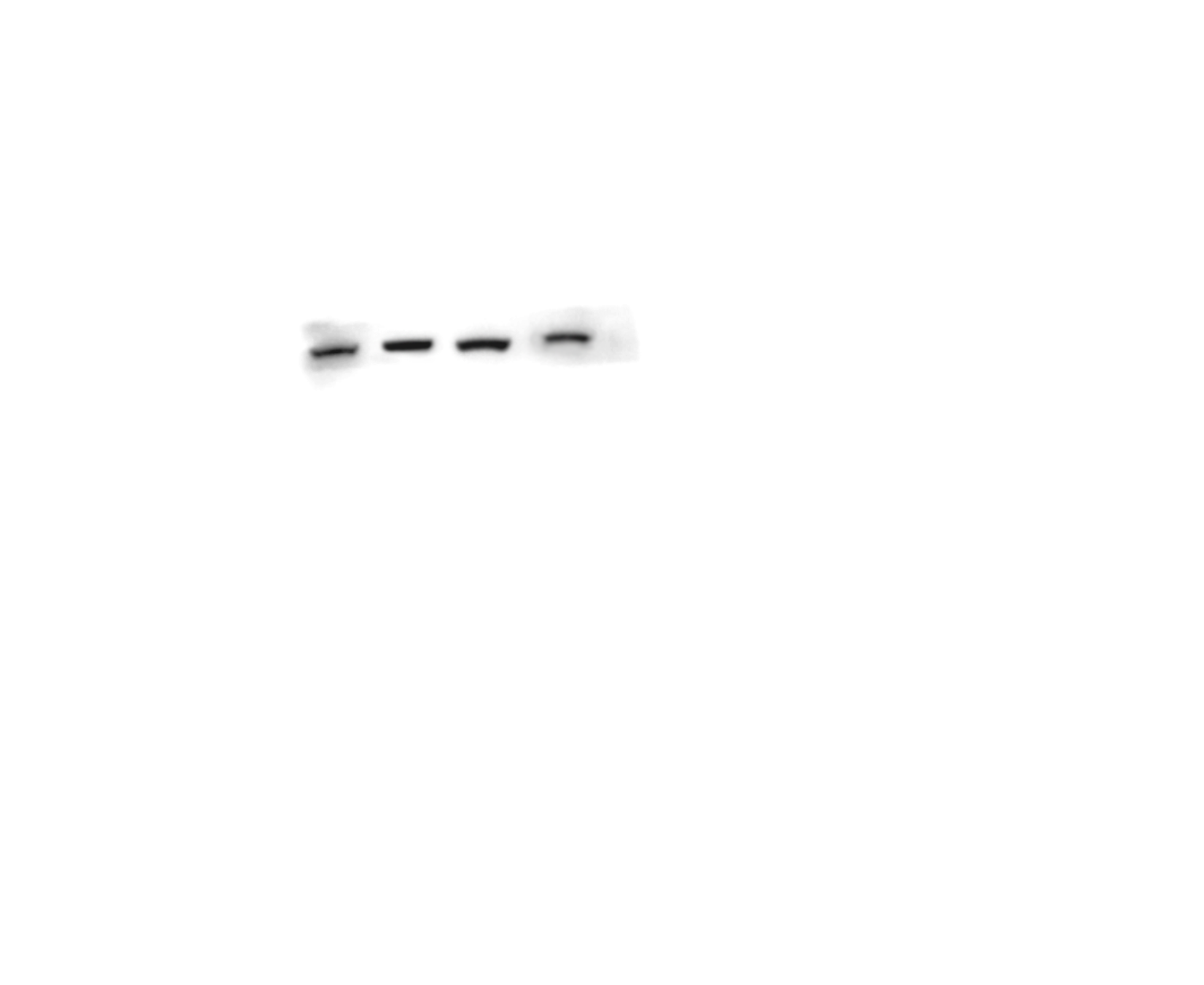


GAPDH (01) GAPDH (02) GAPDH (03)


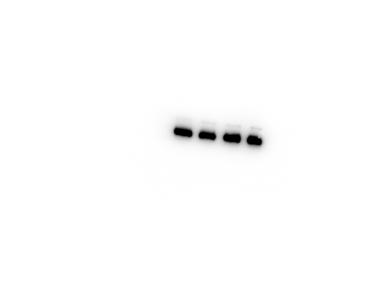

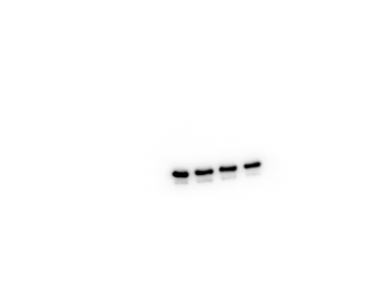

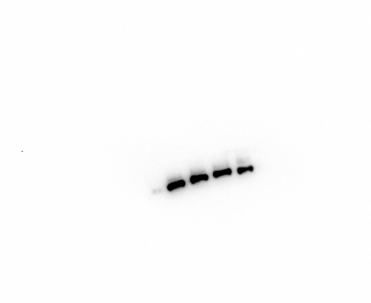


Figure 4D The expressions of TLR4 protein in 16HBE cells transfected with miR-885-3p mimics.

TLR4 (01) TLR4 (02) TLR4 (03)


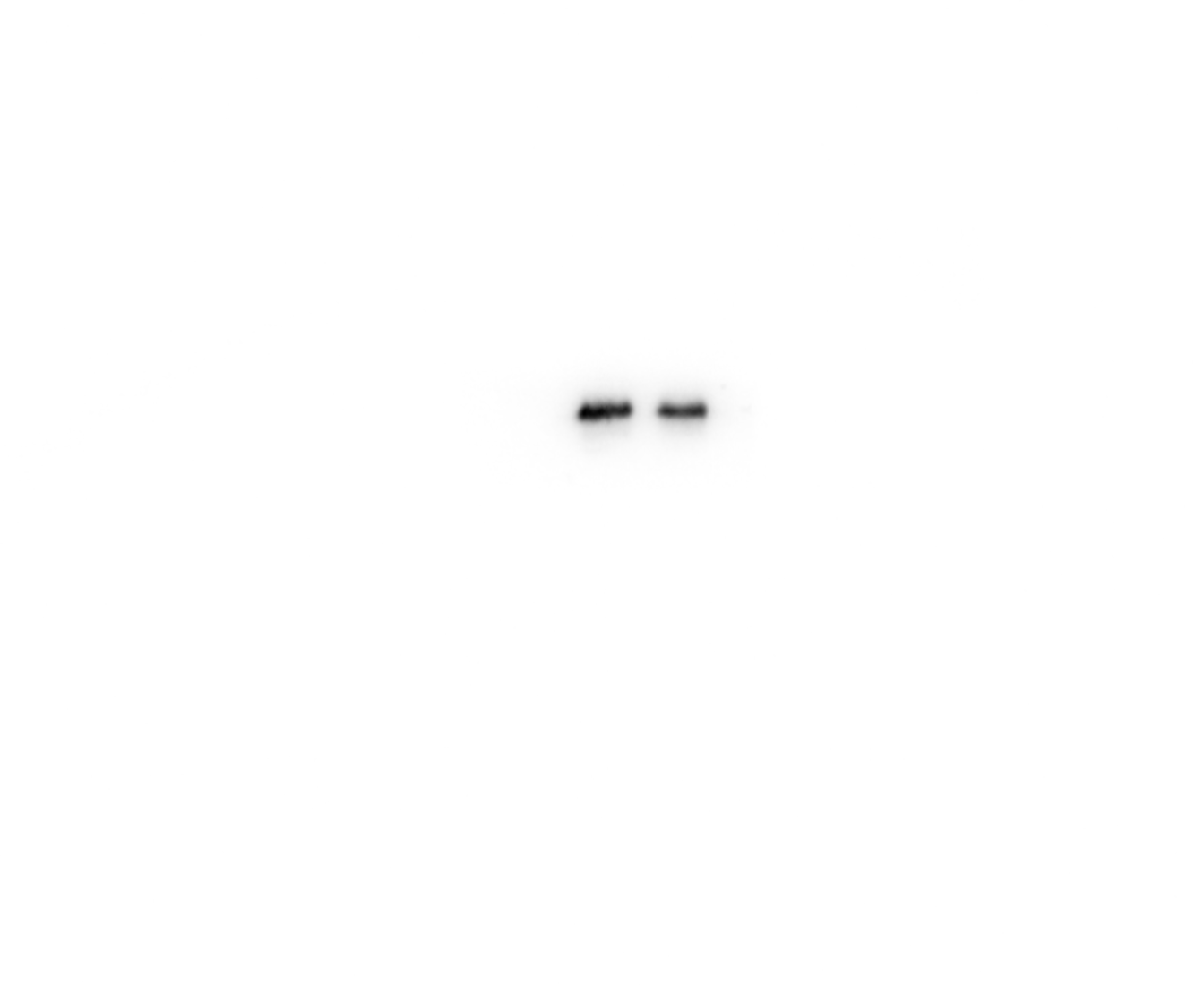

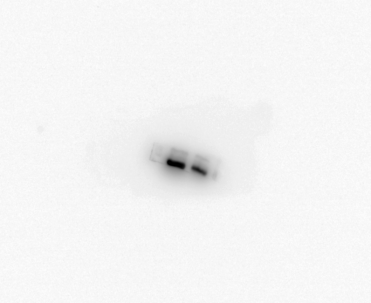

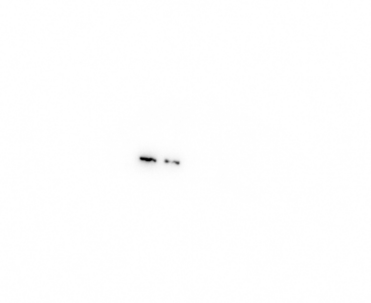


GAPDH (01) GAPDH (02) GAPDH (03)


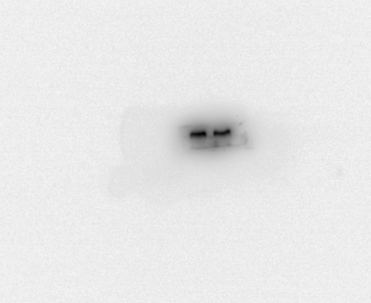

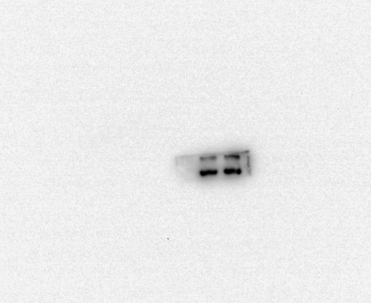

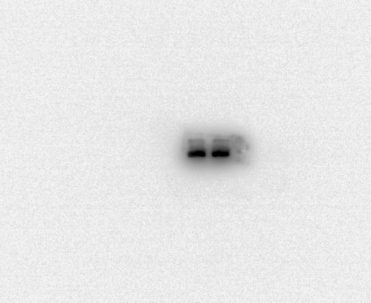


Figure 4F The expressions of TLR4 protein in 16HBE cells stimulated with different concentrations of LPS (5, 10 and 20 μg/ml).

TLR4 (01) TLR4 (02) TLR4 (03)


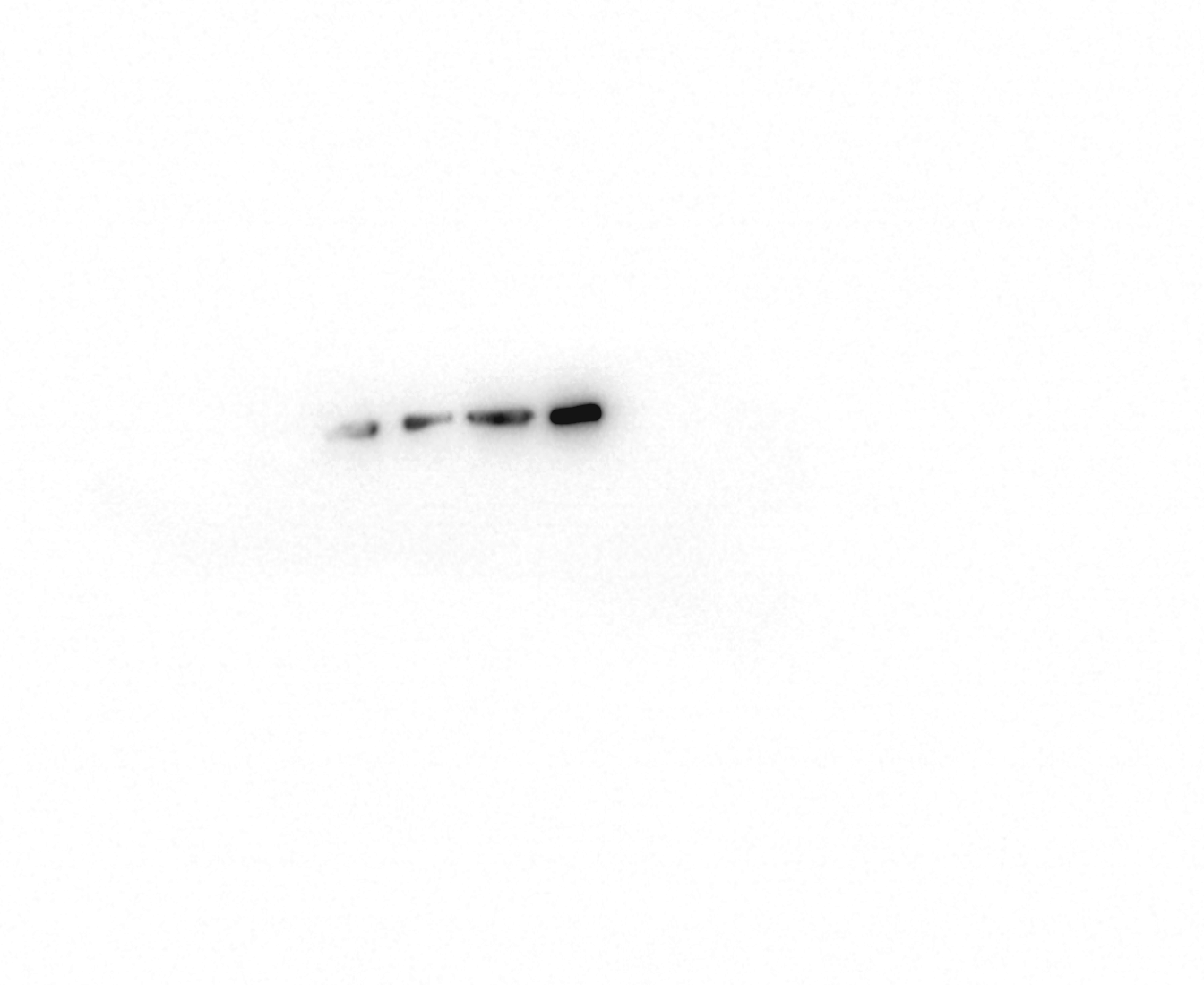

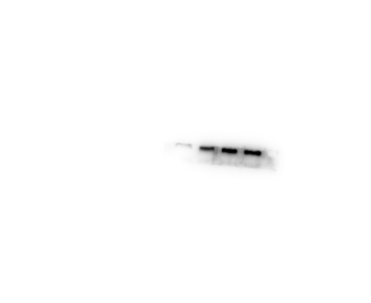

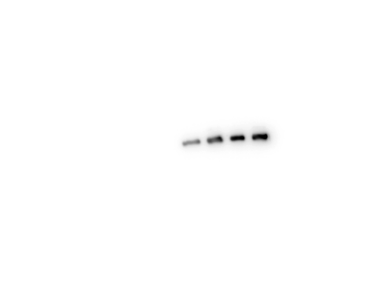


GAPDH (01) GAPDH (02) GAPDH (03)


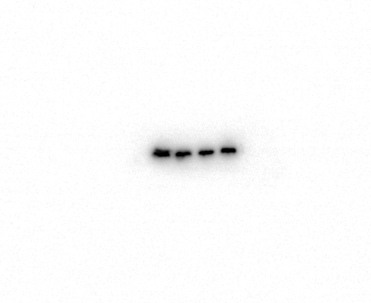

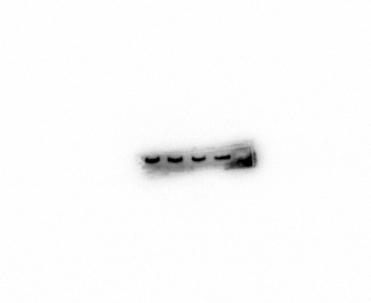

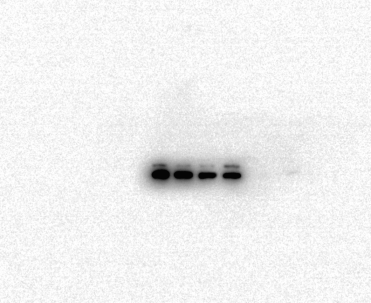


Figure 5B The TLR4 protein expressions in 16HBE cells treated with 10 μg/ml LPS or transfected with miR-885-3p mimics or co-transfected with miR-885-3p mimics and TLR4 overexpression plasmids.

TLR4 (01) TLR4 (02) TLR4 (03)


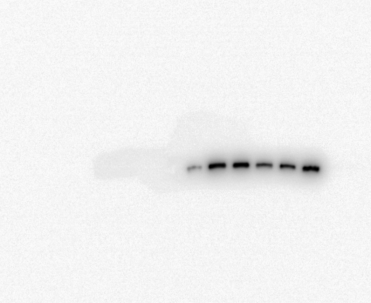

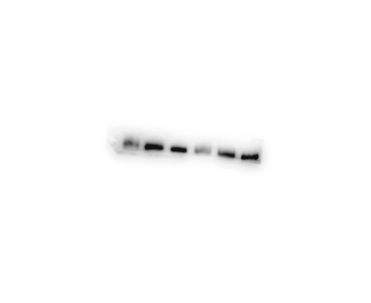

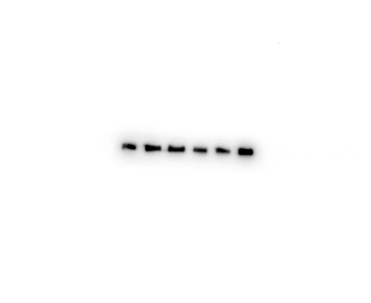


GAPDH (01) GAPDH (02) GAPDH (03)


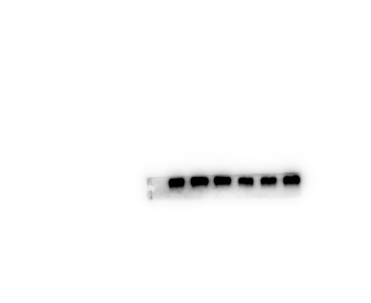

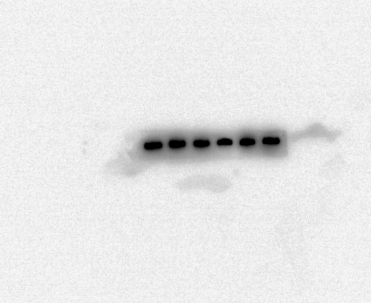

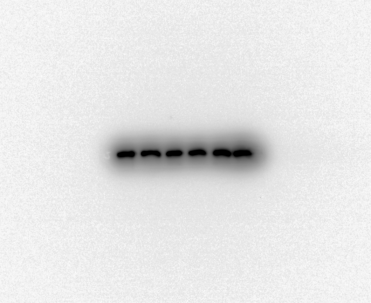


Figure 5H The protein expression of Bcl-2, Bax and cleaved caspase-3 in 16HBE cells treated with 10 μg/ml LPS or transfected with miR-885-3p mimics or co-transfected with miR-885-3p mimics and TLR4 overexpression plasmids.

Bcl-2 (01) Bcl-2 (02) Bcl-2 (03)


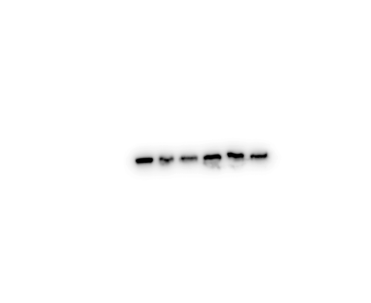

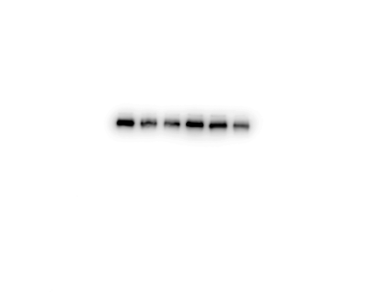

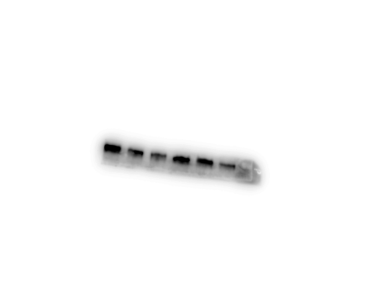


Bax (01) Bax (02) Bax (03)


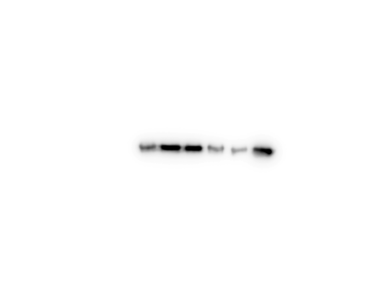

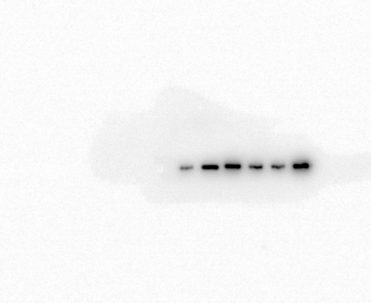

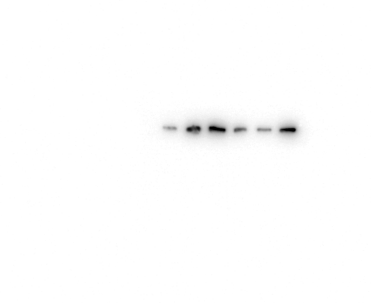


cleaved caspase-3 (01) cleaved caspase-3 (02) cleaved caspase-3 (03)


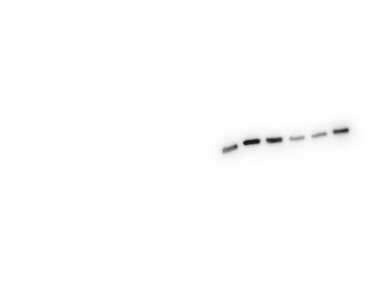

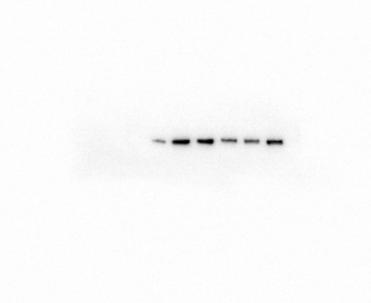

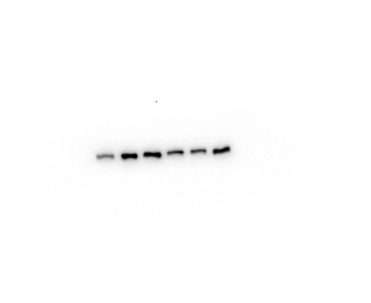


GAPDH (01) GAPDH (02) GAPDH (03)


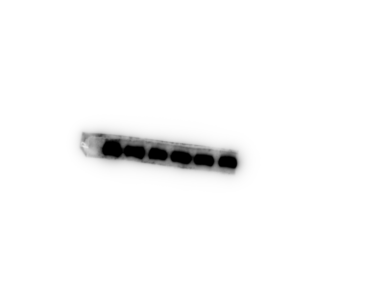

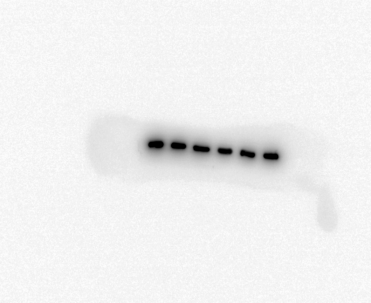

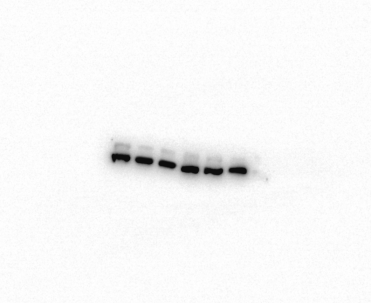


Figure 6A The protein expression of MyD88, NF-κB p65 and p-NF-κB p65 in 16HBE cells treated with 10 μg/ml LPS or transfected with miR-885-3p mimics or co-transfected with miR-885-3p mimics and TLR4 overexpression plasmids.

MyD88 (01) MyD88 (02) MyD88 (03)


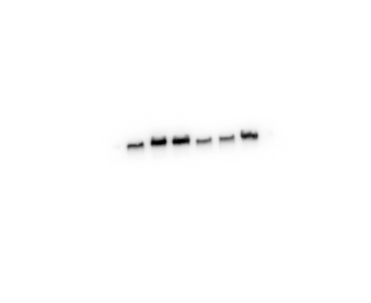

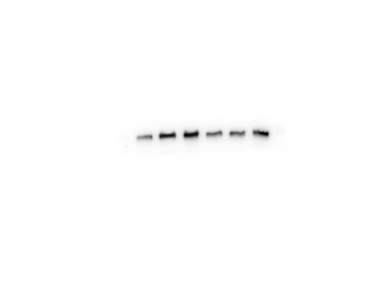

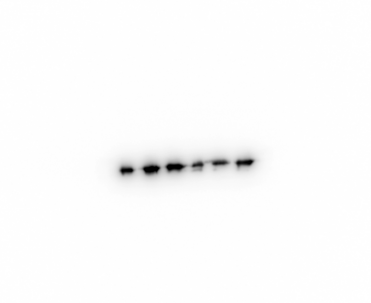


p-NF-κB p65 (01) p-NF-κB p65 (02) p-NF-κB p65 (03)


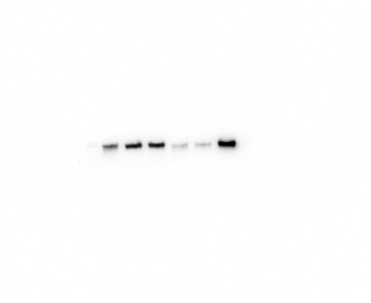

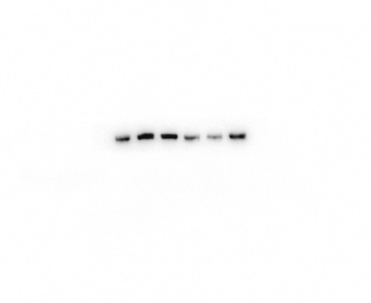

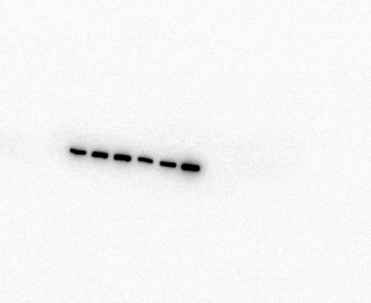


NF-κB p65 (01) NF-κB p65 (02) NF-κB p65 (03)


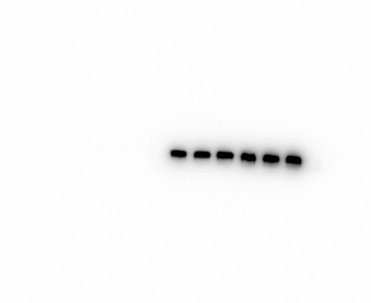

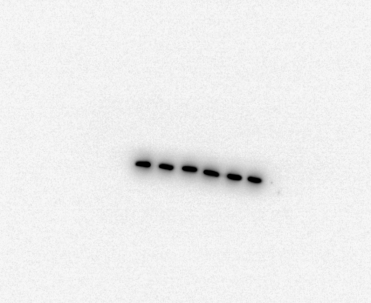

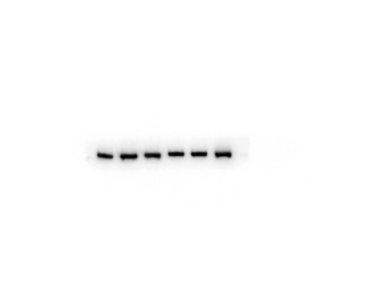


GAPDH (01) GAPDH (02) GAPDH (03)


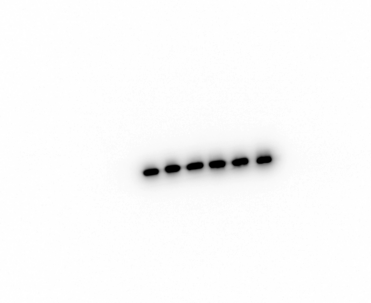

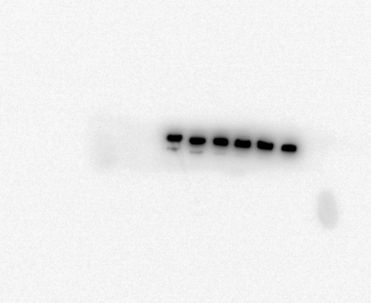

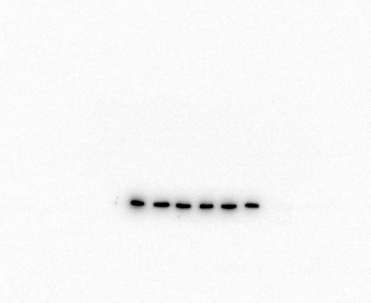

Supplement: Supplemental Material [file KBIE_A_2032939_SM2927.docx]
